# Evaluation of the Relationship Between Age-related Macular Degeneration and Refractive Error, Socio-demographic Features, and Biochemical Variables in a Turkish Population

**DOI:** 10.4274/tjo.97254

**Published:** 2018-10-31

**Authors:** Öznur Gürbüz Yurtseven, Sibel Aksoy, Aysu Karatay Arsan, Yelda Buyru Özkurt, Hatice Kübra Kökçen

**Affiliations:** 1 Gebze Fatih State Hospital, Ophthalmology Clinic, Kocaeli, Turkey; 2 University of Health Sciences, Fatih Sultan Mehmet Training and Research Hospital, Ophthalmology Clinic, İstanbul, Turkey; 3 University of Health Sciences, Kartal Dr. Lütfi Kırdar Training and Research Hospital, Ophthalmology Clinic, İstanbul, Turkey; 4 Ataşehir Modern Medical Center, Ophthalmology Clinic, İstanbul, Turkey

**Keywords:** Axial length, refractive error, risk factors, age related macular degeneration

## Abstract

**Objectives:**

To investigate the relationship between age-related macular degeneration (AMD) and refractive error and axial length, as well as the socio-demographic characteristics and biochemical variables that may affect this relationship.

**Materials and Methods:**

A total of 196 eyes of 98 patients over 50 years of age who were diagnosed with AMD at our clinic were included in this cross-sectional study. Early and late AMD findings were categorized according to the age-related eye disease study grading scale. Objective refractive error was measured by autorefractometer, confirmed by subjective examination, and spherical equivalent was calculated. Refractive errors of -0.50 D to 0.50 D were classified as emmetropia, <-0.50 D as myopia, and >0.50 D as hyperopia. Axial length was measured by ultrasonic biometry and values ≤23.00 mm were classified as short, >23.00 and <24.00 mm as normal, and ≥24.00 mm as long axial length. Demographic, systemic, and biochemical parameters of all patients were also investigated.

**Results:**

Hypermetropic refractive error and shorter axial length were significantly more common than the other groups (p<0.01). No differences were observed between early and late stage groups in terms of refractive error and axial length. Patients with myopia had significantly lower values for total cholesterol, triglyceride, fasting blood glucose, and proportion of smokers. Rates of oral nutritional supplement use and fish consumption were significantly higher in the early AMD group. The most common comorbidity among the AMD patients in our study was essential hypertension.

**Conclusion:**

Hyperopic refractive error and shorter axial length were found to be associated with AMD. Longitudinal studies including larger patient numbers are needed to elucidate the causal and temporal relationship between hyperopic refractive error and AMD.

## Introduction

Age-related macular degeneration (AMD) is the most common cause of central vision loss among individuals aged 55 years and older in both developed and developing countries. The incidence of AMD is increasing due to the growing elderly population, and this constitutes a serious public health problem.^1,2^

AMD has two types, the wet form characterized by neovascularization and the dry form characterized by atrophy. These wet and dry forms account for approximately 20% and 80% of AMD cases, respectively. The wet type is responsible for 85% of AMD-related blindness.^3^ AMD is also clinically classified as early and intermediate stage, which involve drusen and retinal pigment epithelium alterations, or advanced stage, which involves choroidal neovascularization (CNV) and/or geographic atrophy (GA).^4^

Today, AMD is considered a multifactorial disease associated with genetic and environmental factors. Age is the strongest non-modifiable risk factor. The risk of developing advanced AMD is 3 times higher among individuals aged 60-80 years than in those under the age of 60.^5^ Smoking is another important but modifiable risk factor. Many studies have demonstrated the impact of smoking on AMD development and report that smokers are likely to develop AMD 5-10 years earlier than non-smokers.^6^ Epidemiological studies have reported that AMD may be associated with genetics, family history, obesity, low education level, diet, history of cardiovascular and cerebrovascular disease, exposure to sunlight, and various other factors.^7,8,9,10,11,12,13,14^ Possible associations between AMD and ocular factors such as light iris color, history of previous cataract surgery, short axial length, and hypermetropic refractive error have also been proposed.^15,16^ However, there are inconsistencies among the literature data, and no studies have been conducted previously in the Turkish population. 

Understanding how refractive error and axial length are related to AMD may elucidate its pathophysiology and lead to the development of new diagnostic and therapeutic options. The aim of this study was to examine the relationship between AMD and refractive error and axial length, and to investigate the systemic and demographic characteristics that may affect it.

## Materials and Methods

This prospective study was approved by the Scientific Research Commission of Fatih Sultan Mehmet Training and Research Hospital with approval number 17073117-050.03-2268 on September 3, 2013 and was conducted in accordance with the principles of the Declaration of Helsinki. Written informed consent forms were obtained from all patients. The study included 196 eyes of 98 patients who presented to our clinic between October 2013 and June 2014 and were diagnosed with AMD. All patients in the study underwent a complete ophthalmologic examination. Diagnosis of AMD was based on findings of biomicroscopic dilated fundus examination, optical coherence tomography (NIDEK RS-3000 Advance), and fluorescein angiography. AMD lesions were assessed from color fundus images and classified as follows according to the age-related eye disease study (AREDS) staging system.^7^

**Category 1: **No drusen or a few small drusen in both eyes.

**Category 2:** Extensive small drusen, a few intermediate-sized drusen, or pigmentary abnormalities associated with AMD in at least one eye.

**Category 3:** One or more large drusen or extensive intermediate-sized drusen in at least one eye.

**Category 4:** GA or CNV in at least one eye.

Patients evaluated as category 1 or 2 were classified as early AMD and patients in categories 3 and 4 were classified as advanced AMD.

Patients with ocular disease other than AMD or pterygium and/or nuclear cataracts that could affect refractive error; aphakic or pseudophakic patients; anisometropic patients; and patients with history of refractive or any other ocular surgery other than intravitreal injection were excluded from the study. 

Objective refractive error was measured with an autorefractometer (Canon RK-F1 full auto ref-keratometer, Tokyo, Japan) and confirmed by subjective examination. Spherical equivalent refraction was calculated in diopters (D) by adding half of the cylindrical value to the spherical value. Values between +0.50 D and -0.50 D were defined as emmetropia, values below -0.50 D as myopia, and values above +0.50 D as hypermetropia. Axial length was measured with an ultrasonic biometry (NIDEK US-4000 Echoscan, Japan) device; values of 23 mm and below were assessed as short, values between 23 and 24 mm as normal, and values of 24 mm and above as long.

Data pertaining to the patients’ sex, age, systemic comorbidities (diabetes mellitus, hypertension, hyperlipidemia), smoking history (pack-years), fish consumption (meals/month), use of oral nutritional supplement (ONS) (multivitamin and mineral supplement containing 5-10 mg of lutein and zeaxanthin, tablets/day), use of acetyl salicylic acid (ASA), and body mass index were recorded. In addition, lipid panel (total cholesterol, triglycerides [TG], low-density lipoproteins [LDL], high-density lipoproteins [HDL]), fasting blood sugar (FBS), and hemoglobin A1c (HbA1c) levels were assessed for all patients.

### Statistical Analysis

The NCSS (Number Cruncher Statistical System) 2007 software and PASS (Power Analysis and Sample Size) 2008 Statistical Software (Utah, USA) were used for statistical analyses. For quantitative data, descriptive statistical methods (mean, standard deviation, median, frequency, ratio, minimum, maximum) were used as well as Student’s t-test for pairwise comparisons of parameters with normal distribution and Mann-Whitney U test for pairwise comparisons of parameters without normal distribution. A one-way ANOVA was used for the comparison of normally distributed data between three or more groups and Tukey HSD test was used to determine the source of the difference. A Kruskal-Wallis test was used for the comparison of non-normally distributed data between three or more groups and Mann-Whitney U test was used to determine the source of the difference. Pearson’s chi-square test, Fisher’s exact test, Fisher-Freeman-Halton exact test, and Yates’ correction for continuity (Yates’ corrected chi-square test) were used in comparisons of qualitative data. Significance was evaluated at p<0.01 and p<0.05.

## Results

The study included 50 female and 48 male patients with a mean age of 70.18±6.90 (54-85) years. Of these, 85.8% of the patients had a low education level, 46.9% were smokers, 97.8% consumed fish, 44.7% used ONSs, 30.6% used ASA, and 71.4% had a comorbid systemic disease. The most common systemic comorbidity was hypertension. The patients’ demographic characteristics are presented in Table 1 and their biochemical data in Table 2. 

In terms of refractive status distribution, 10.2% of the patients were myopic, 18.4% were emmetropic, and 71.4% were hypermetropic. Hypermetropia was significantly more common than the other groups (p<0.01). Refractive values ranged between +0.50 D and +3.00 D in 94.3% of hypermetropic patients, while 5.7% of patients had values higher than +3.00 D. The refractive error rates of the patients who participated in the study are given in Table 3.

Short axial length was noted in 83.7% of the patients, which was significantly more common than normal or long axial length (p<0.01). The axial length rates of the patients who participated in the study are given in Table 4.

Evaluation of biochemical parameters based on refractive error revealed statistically significant differences in cholesterol and TG values (p<0.05). Paired evaluations done to determine the source of the difference showed that patients in the myopia group had significantly lower total cholesterol levels compared to patients in the hypermetropia group (p=0.045). There was no statistically significant difference between the emmetropia and hypermetropia groups (p>0.05). Patients in the myopia group had significantly lower TG values than patients in both the emmetropia and hypermetropia groups (p=0.014, p=0.001). There was no statistically significant difference between the emmetropia and hypermetropia groups (p>0.05). Analysis of FBS values in the refractive error groups showed a difference that was near statistical significance (p=0.058). According to paired evaluations, patients in the emmetropia and myopia groups had significantly lower FBS than patients in the hypermetropia group (p=0.021). There was no significant difference between the emmetropia and myopia groups (p>0.05). There were no statistically significant differences in HDL, LDL, or HbA1c values based on refractive error (p>0.05). The distribution of biochemical parameters based on refractive error is shown in Table 5.

There was a significant difference between the refractive error groups in the proportion of smokers (p=0.001, p<0.01). The rate of smoking was statistically significantly lower in the myopia group than in both the emmetropia and hypermetropia groups. ASA use was also significantly less common in the myopia group compared to the emmetropia and hypermetropia groups (p=0.011). No statistically significant differences were observed between the refractive error groups in terms of fish consumption, ONS use, or comorbidities (p>0.05).

Evaluations based on AMD stage revealed no statistically significant differences in terms of mean axial length, refractive error, total cholesterol, HDL, LDL, TG, FBS, HbA1c values, smoking, ASA use, or comorbidities (p>0.05). Fish consumption and ONS use were significantly more common among patients with early AMD compared to those with advanced AMD (p=0.046, p=0.001). Distributions of axial length, refractive error, biochemical parameters, and usage habits based on AMD stage are presented in Table 6.

## Discussion

In this study, the relationship between refractive error and AMD was investigated in a Turkish population and a strong correlation was found between AMD and the prevalence of hypermetropic refractive error. The literature includes numerous cross-sectional studies and a few longitudinal studies that investigate the relationship between hypermetropia and AMD. 

In the Beijing Eye study, conducted in an Asian population, it was reported that hypermetropia is the most significant risk factor for early AMD, independent of age.^17^ In another study conducted on Asian multiethnic groups, the prevalence of AMD was lower in myopic males, but there was no increased risk in those with hypermetropia.^18^ In the Rotterdam study, conducted in a white population, the prevalence of hypermetropia was found to be 65% and every 1 mm of decrease in axial length was associated with an increase in the incidence and prevalence of AMD.^19^ The Eye Disease and AREDS cross-sectional case-control studies reported 1.5 and 2.3 times more exudative AMD in hypermetropic patients compared to myopic patients after correcting for age and other risk factors.^4,20^

According to the Singapore Malay Eye study, also conducted in an Asian population, every 1 D increase in refractive error and every 1 mm decrease in axial length increased the risk of early AMD by 8% and 29%, respectively. A similar relationship was not found for advanced AMD and it was reported that this could be due to the smaller number of patients with late stage disease.^21^ In a 5-year longitudinal follow-up of the same group, this relationship between early AMD and refractive error was not apparent.^22^ Similarly, a cross-sectional study conducted by the Blue Mountains Eye Study group showed a correlation between moderate and high hypermetropia values and the incidence of early AMD, but a longitudinal study involving the 5-year follow-up of the same patients revealed no significant correlation between hypermetropia and AMD incidence.^23,24^ In the Beaver Dam Eye study, 5-year and 10-year follow-up also failed to show any correlation between refractive error and the incidence of AMD.^25,26^

It appears that the correlations between AMD and hypermetropic refractive error observed in cross-sectional studies are not found in longitudinal studies. Some patients are lost over a follow-up period of 5-10 years, and therefore the lifespan factor may alter the outcomes of longitudinal studies. For example, the Blue Mountains group stated in their study that most of the patients who died during follow-up were hypermetropic, and that the results may have been different if these patients had survived.

In our study, the prevalence of hypermetropia was 71.4% among all AMD stages and 72% and 68% for early and advanced AMD, respectively, while the prevalence of myopia was found to be 10% among all AMD stages and in both early and late AMD. Short axial length was noted in 83.7% of our patients. The results of our study are comparable to those of the Singapore Malay Eye, Beijing Eye, Rotterdam, Blue Mountains, Eye Disease, and AREDS groups. The results of these studies suggest that hypermetropia generally increases the risk of early AMD but is not associated with a significant increase in the risk of late AMD. In our study, however, there was no significant difference between the early and late AMD groups in terms of short axial length and hypermetropic refractive error. This may be attributable to the insufficient size of our patient group, which is the main limitation of our study. There are several possible biological explanations for the relationship between refractive error, axial length, and the pathogenesis of AMD. Hypermetropic eyes with short axial length have greater scleral rigidity. This creates resistance in choroidal venous outflow, and reduced outflow may contribute to the development of AMD due to the accumulation of metabolic waste.^27,28,29^ Vascular endothelial growth factor (VEGF) plays a key role in the pathophysiology of AMD. According to recent findings, intraocular VEGF level decreases as degree of myopia and axial length increase.^30,31^ Longer axial length may lead to increased VEGF dilution and lower risk of disease. Myopic eyes are prone to posterior vitreous detachment (PVD).^32,33^ PVD has been shown to reduce the progression of neovascularization in diabetic eyes.^34^ Considered from this perspective, PVD may exert a protective effect against AMD through increased oxygen diffusion in the macular region. On the other hand, glasses and contact lenses used by myopic patients may reduce exposure to ultraviolet radiation, which is recognized as a significant risk factor in the etiology of AMD.^13,14,35^

The lower prevalence of AMD in the myopic patient group in our study may have been due to their lower cholesterol, TG, and FBS levels and lower smoking rate. Our comparison of the early and advanced disease groups revealed significantly higher rates of fish consumption and ONS use in the early disease group. This offers further evidence of the positive effect of antioxidant fatty acids and vitamins such as omega-3, lutein-zeaxanthin, and vitamins A, C, and E, which have been emphasized as important components of preventative treatment in many studies.

## Conclusion

Our study showed short axial length and hypermetropic refractive error to be associated with AMD, independent of demographic and systemic findings. The major limitations of our study are its cross-sectional design and the small number of patients. The small patient number reduces the power of the study. Cross-sectional studies cannot demonstrate the temporal and causative relationship between a factor and an outcome. AMD itself may also cause changes in refractive status and axial length. Examining for and questioning ocular, systemic, and environmental factors in patients over the age of 50 is beneficial for early diagnosis and follow-up as well as providing opportunities for preventive therapy and the modification of environmental factors.

## Figures and Tables

**Table 1:**
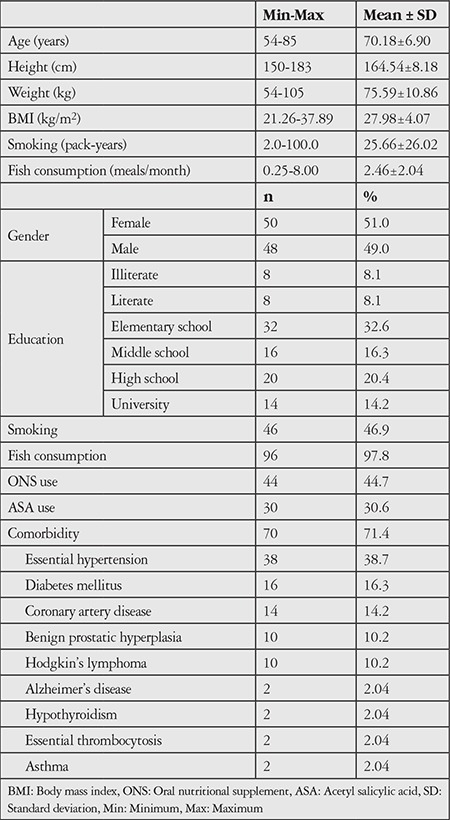
Demographic characteristics of the patients

**Table 2:**
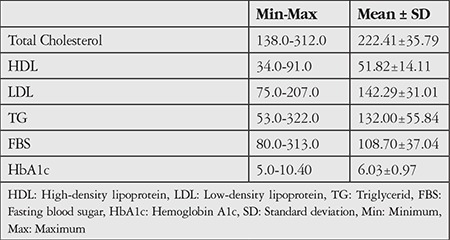
Distribution of biochemical variables

**Table 3:**
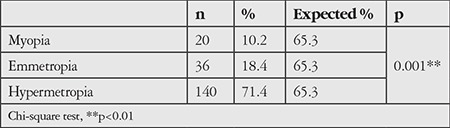
Refractive error rates

**Table 4:**
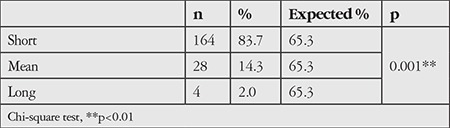
Axial length rates

**Table 5:**
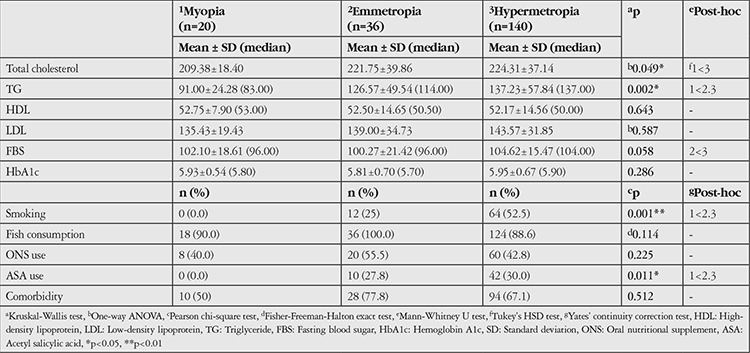
Evaluations based on refractive error

**Table 6:**
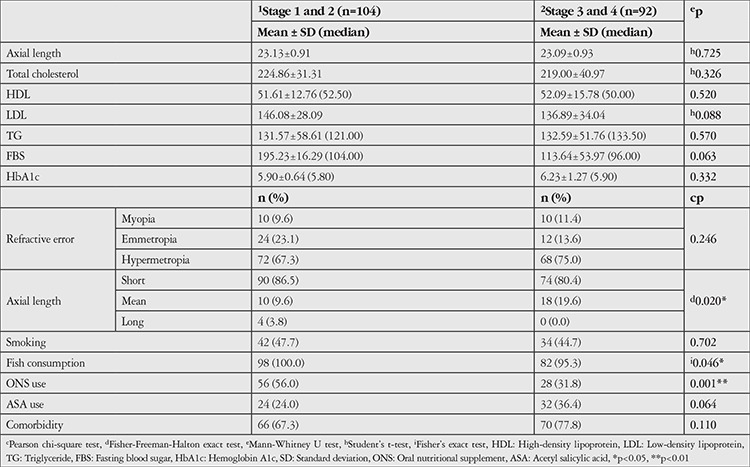
Evaluations according to stage of age-related macular degeneration
